# Transferring embryos with indeterminate PGD results: the ethical implications

**DOI:** 10.1186/s40738-016-0014-9

**Published:** 2016-02-01

**Authors:** Iris G. Insogna, Elizabeth Ginsburg

**Affiliations:** 1 Department of Obstetrics & Gynecology, Brigham & Women’s Hospital, 75 Francis Street, Boston, MA 02115 USA; 2 Department of Reproductive, Endocrinology and Infertility, Brigham & Women’s Hospital, 75 Francis Street, Boston, MA 02115 USA

**Keywords:** Ethics, Reproductive ethics, Pre-implantation genetic diagnosis

## Abstract

**Background:**

In vitro fertilization (IVF) and pre-implantation genetic diagnosis (PGD) are becoming increasingly common techniques to select embryos that are unaffected by a known genetic disorder. Though IVF-PGD has high success rates, 7.5 % of blastocysts have inconclusive results after testing. A recent case involving a known BRCA-1 carrier was brought before our Assisted Reproductive Technology Ethics Committee in order to gain a better appreciation for the ethical implications surrounding the transfer of embryos with indeterminate testing.

**The case presentation:**

Thirty-nine year old G0 BRCA-1 carrier requiring IVF for male factor infertility. The couple elected for PGD to select against BRCA-1 gene carrier embryos. However, several embryos were returned with inconclusive results. The couple wished to proceed with the transfer of embryos with an unknown carrier status. The case was presented before our Assisted Reproductive Technology Ethics Committee.

**Conclusion:**

Many considerations were explored, including the physician's duty to protect patient autonomy, the physician's duty to act in the best interest of the future child, and the physician's duty towards society. Transferring both embryos with unknown carrier status and known-carrier status was debated. Ultimately, the transfer of inconclusive embryos was felt to be ethically permissible in most cases if patients had been adequately counseled. However, the re-biopsy of embryos with inconclusive testing results was encouraged. The transfer of known-carrier embryos was felt to be unethical for certain disease-states, depending on the severity of illness and timing of disease onset. We strongly encourage physicians to create an action plan in advance with their patients, prior to testing, in the event that embryos are returned with inconclusive PGD results. The committee’s decision, though helpful in guiding practice, should not overshadow the individual physician-patient relationship, and the need for thorough counseling.

## Background

In vitro fertilization (IVF) and pre-implantation genetic diagnosis (PGD) are becoming increasingly common techniques to select embryos that are unaffected by a known genetic disorder. Couples may opt for PGD if they are carriers of the gene for Tay-Sachs or Huntington’s disease, for example, conditions with well-understood and predictable patterns of inheritance. PGD is also increasingly desired by patients with hereditary cancer syndromes. A recent meta-analysis of 13 studies which included 370 respondents affected by a hereditary cancer syndrome, reported that 28 % felt their syndrome impacted family planning, 72 % felt that PGD should be offered, and 43 % would consider using PGD [1]. Acceptance of PGD has been well-studied in patients specifically affected by hereditary breast and ovarian cancer (HBOC) as well [2]. In a survey of 22 couples affected by HBOC, half chose to undergo PGD because they “believed it was their moral duty to protect their future child(ren) from suffering” [3].

PGD, however, is an imperfect science, and does not always provide conclusive results [4, 5]. Though published data report that misdiagnosis is rare, with rates of <1 % for polymerase chain reaction (PCR) cases and <5 % for fluorescence in situ hybridization (FISH) cases, some studies suggest that misdiagnoses may be underestimated or underreported [6]. Approximately 7.5 % of blastocysts have inconclusive results after testing [7]. Indeterminate results in PGD, and the development of pregnancies affected by a genetic disorder that failed to be detected, can have long-lasting and devastating effects.

When testing is indeterminate, the IVF team is confronted with several ethical considerations regarding whether or not to proceed with embryo transfer. Many perspectives must be weighed under these circumstances. Employing the founding principles of bioethics, as laid out by Beauchamp and Childress, are effective means of clarifying the ethical stakes involved [8]. These include considering the patient’s right to autonomy, particularly reproductive autonomy, the physician’s responsibility of beneficence towards her patient, the physician’s duty of non-maleficence regarding the unborn child he or she is helping to create, and the implications for society at large, a broader question hinging on the principle of justice. A recent case at our institution, presented below, compelled us to examine these considerations in greater depth.

## Case presentation

In the following case, all potential patient information was de-identified, consent was not necessary or requested, and the case was exempt from IRB approval. Patient S was a 39-year-old Gravida 0 and was a known BRCA-1 gene carrier, status post prophylactic bilateral mastectomy. After trying to conceive for over a year, she and her husband underwent an infertility evaluation, which revealed severe male factor infertility, prompting them to pursue IVF and consider PGD.

BRCA-1 mutations have autosomal dominant inheritance. Women who carry the gene have a 57 % lifetime risk of developing breast cancer and 40 % risk of developing ovarian cancer by the age of 70 [9]. Male BRCA-1 carriers have a lifetime breast cancer risk of less than 2 %, but may be twice as likely to develop prostate cancer before age 65, and this may be aggressive and be associated with poorer survival when compared to the general population [10]. Diligent screening practices, and prophylactic surgeries in known carriers, reduce but do not eliminate these risks. Having already undergone prophylactic bilateral mastectomy, our patient planned to undergo a risk reducing prophylactic bilateral salpingo-oophorectomy at age 40.

Given her BRCA-1 status, and the indication for assisted reproductive technology to treat her infertility, the patient and her husband opted to undergo IVF/ ICSI (intracytoplasmic sperm injection) and PGD to select non-carrier embryos for BRCA-1, and wished to transfer two unaffected embryos. Pre-implantation genetic screening for chromosome evaluation was offered but was not covered by her insurance, and the couple deferred this testing. As with all routine obstetric care, post-conception genetic screening and confirmatory testing would be offered should the couple so choose.

The IVF/ICSI cycle resulted in 17 oocytes retrieved and 10 fertilized. Of those, 5 day 5 blastocysts underwent biopsy and PGD with the following results: one affected embryo, one unaffected embryo, one embryo with no results (no DNA amplification), and two embryos with inconclusive test results. The embryos with inconclusive results were Grade 5 BC (blastocysts with poor trophectoderm cells). Data suggest that twice-frozen embryos have statistically significant lower post-thaw survival rates compared to once-frozen embryos (87.5 % vs 98.3 %) but have equivalent pregnancy and live birth rates [11, 12]. Generating an inconclusive result is an intrinsic risk in PGD. A 2013 study estimated that for blastocyst biopsy, cryopreservation and thawed embryo transfer, the diagnostic rate is 90 % with 5 % amplification failure and 5 % allele drop-out [4].

Despite the frustrating outcome of the cycle, the decision was made to proceed with the transfer of the one unaffected embryo. The patient, however, expressed a strong desire to transfer the embryos with inconclusive results if the initial transfer was unsuccessful, and declined re-biopsying the embryos.

Currently, there exist no overarching guidelines dictating what a physician should do regarding embryos with inconclusive results. As initially the patient opted for PGD in order to avoid transmission of the BRCA-1 gene to her future child, it seems inconsistent to then transfer an embryo with an unknown genotype that has a 50 % chance of being a carrier. It is difficult to predict the individual likelihood of developing a future malignancy, as the BRCA-1 gene displays incomplete penetrance. While many infertility specialists agree there is a professional “obligation to not (deliberately) transfer an affected embryo,” there is no consensus regarding embryos with indeterminate carrier status [13]. The European Society of Human Reproduction and Embryology (ESHRE) comments that physicians, as “collaborators in the parental project” ought to refuse transfer of an embryo that may be affected by a condition that poses a “high risk of serious harm to the future child.” The ESHRE, however, offers no concrete definition of the term “serious harm” [14].

The patient’s request to transfer an embryo with inconclusive testing raises the question of where the physician’s primary responsibility lies. The physician has a Hippocratic duty to his or her patient and to protecting that patient’s reproductive autonomy, but there are conflicting duties that complicate the decision-making process. The physician must consider his or her duty towards the future child, and potentially his or her responsibilities towards society in general. Is it a poor and inappropriate allocation of societal resources to assist in the creation of an adult at high risk of requiring extensive financial and/or psychological support, morbidity and possible mortality?

### “Results: the deliberation of the Ethics Committee”

Currently, fertility specialists confronted by such scenarios are compelled to make decisions on a case-by-case basis.

Given its complexity, the case of Patient S was brought before the department’s Assisted Reproductive Technology (ART) Ethics Committee. The committee includes attending physicians, fellows, residents, a bioethicist, embryologists, nurses, a psychiatrist, social workers, attorneys, and the head of the hospital IRB. Early in the discussion, some members argued in strong support of patient autonomy, feeling compelled to complete the transfer of indeterminate embryos granting the couple the ultimate decision-making power over the fate of their embryos. Similarly, it was argued that the transfer of an indeterminate embryo should be allowed in this case if the couple’s primary concern was to have a child. If the couple was willing to acknowledge and accept the risk of having an affected child with a BRCA-1 gene, then the transfer was acceptable.

Other members felt strongly that the transfer should be prohibited, given that the objective of PGD was to avoid the transmission of known deleterious genes, and that re-biopsy and analysis of the embryo prior to transfer should be mandatory, even though removal of more trophectoderm cells might reduce the chance of the embryo implanting. They argued that if the couple’s primary concern was to have a child, regardless of that child’s carrier status, then they should not have undergone PGD at all.

There was also significant debate about how to define the appropriateness of transfer of indeterminate embryos when considering different diseases. For example, members seemed to voice stronger opinions against transferring an embryo that was a potential Tay-Sachs carrier than one with the potential to be a BRCA-1 carrier. Members argued that transferring an embryo possibly affected by an immediately fatal condition would never be in the future child’s best interest. However, when considering less debilitating or non-fatal diseases, they might be more willing to consider transfer of indeterminate embryos. No consensus could be reached, however, regarding how to categorize disease states as more or less debilitating; this discussion is ongoing and involves members of the broader hospital ethics committee.

The committee concluded that the transfer of known *affected* embryos should be prohibited, and that this policy be accepted by the couple prior to treatment, based on the principle that if a patient is willing to accept an affected embryo, then PGD is unnecessary. There was no definitive decision regarding indeterminate embryos, however.

What follows is an examination of the committee’s initial decision through a bioethical lens. The stakes involved in this case require careful consideration of patient autonomy, non-maleficence, justice.

### Discussion part 1

#### Protecting patient autonomy

The couple’s request was understandable, and founded in an intense desire for a biological child. They had invested substantial resources to proceed with the IVF cycle, financially, emotionally and physically. Their treatment required multiple clinic visits, blood testing, and potentially risky ovarian stimulation and oocyte retrieval. The cost burden of infertility is significant, and often impacts the decision-making process of patients undergoing treatment. Moreover, undergoing IVF delayed her prophylactic salpingo-oophorectomy, putting her at risk of developing an ovarian malignancy. Her oncologist had strongly encouraged her to proceed with surgery, and recommended against a second IVF cycle to generate more embryos. Pregnancy rates with IVF-PGD are estimated to be 44–50 % [15]. Patient S thus viewed this as her only chance at having a biological child who would not face a medical course similar to her own.

The stakes were clearly very high for this couple. The physician, therefore, had a great responsibility to protect the patient’s reproductive autonomy. The couple has an inalienable right to reproduce, parent, and create a family. By transferring only the one unaffected embryo, and discarding the indeterminate and affected embryos, the patient’s chance of achieving pregnancy could have been significantly compromised.

One might argue that the patient had alternatives to consider, and that she could achieve future pregnancy with a donor egg, for example. However, this patient had a strong desire to conceive and deliver an infant that was biologically hers, so egg donation was out of the question. Alternatively, she could have opted to delay bilateral salpingo-oophorectomy further in order to undergo another IVF cycle, but this was considered too great a risk to her future health. This IVF cycle then may very well have represented her only chance of having a biological child; the physician’s refusal to accommodate her wishes may have threatened her inherent right to reproductive autonomy.

When protecting patient autonomy, it bears mentioning that the embryos in question are the property of the intended parents. Therefore, one might argue the parents should be the sole decision makers regarding the fate of these embryos. If the institution that created the embryos refuses to follow the parents’ wishes, then it is within their right to seek care elsewhere.

### Discussion part 2

#### Protecting the interests of the future child: non-maleficence

A second consideration is the physician’s duty towards the future child, a duty that corresponds to the principle of non-maleficence or “do-no-harm.” The European Society of Human Reproduction and Embryology (ESHRE) Task Force states: “The physician carries joint responsibility for the welfare of the child because of his or her causal and intentional contribution to the parental project. The physician must take into account presently known risk factors for the welfare of the future child. To avoid prejudice, arbitrariness and discrimination, objective evidence must be sought to be able to offer good reasons for refusing assistance” [14].

The Ethics Committee opinion on PGD from the American Society of Reproductive Medicine references the non-identity argument, a philosophical line of reasoning that argues when considering an act that results in the creation of a life, the benefits of having any existence at all, even if that existence is imperfect, may outweigh the potential harm of creating a life that is flawed [16]. This can be extrapolated to argue that it is impossible to harm a being that has yet to exist.

Therefore, the physician must consider carefully the wellbeing of the child he or she is helping to create. The ESHRE Task Force comments that physicians should only refuse to assist patients in their reproductive efforts if “the quality of life of the future child is so low that it would have been better off not to have been born” [14].

This statement seems intuitive, yet its inherently subjective nature requires physicians to make personal judgments regarding their perceived quality of life associated with any given disease state. This is a highly subjective task that is undeniably influenced by personal experience.

When considering disease-states, selecting against certain conditions is more intuitive than others. For example, most people would likely select against transferring embryos that may be affected by Tay-Sachs, a condition inevitably fatal early in life. A counter-example often referred to in the bioethics classroom, is that of congenital deafness. Consider a deaf couple that would like to select for a deaf child. Since deafness is largely considered a disability in our society, in attempting to act in the best interest of the future child a physician might refuse the parents’ request, assuming the future child would prefer to be hearing rather than deaf. However, some deaf couples would argue that deafness is not a disability. Moreover, a hearing child might find living within a deaf community much more challenging, and may very well feel more ostracized than if he were deaf.

Disability rights advocates understandably take issue with the idea of selecting against conditions that have become labeled as disabilities. Selecting against future children affected by Down’s syndrome, blindness, and other inherited conditions currently defined as disabilities may compromise society’s diversity, and limit our acceptance of “otherness.” The use of PGD may risk devaluing certain populations.

Only approximately “three percent of American IVF-PGD clinics report having provided PGD to couples who seek…to select an embryo for the presence of a disability” [17]. Again, there is no consensus on the definition of disability. Advocates of disability rights may argue, regardless of the label, that is it discriminatory to categorically select against all inherited conditions that fall outside of conventional concepts of normal.

In the current case, if the indeterminate embryo produces a child that carries the BRCA-1 gene, what does that imply for the child’s quality of life? One future adult may feel devastated by the implications of being a carrier of this gene, while another may find it hardly a burden at all. Hereditary breast and ovarian cancer syndromes, along with many other incompletely penetrative diseases, lead to a more subtle deliberation regarding the future child’s “best interest.” Not every BRCA-1 gene carrier develops cancer. Of those that do develop cancer, not all succumb to the disease. Prophylactic surgery dramatically reduces mortality. For BRCA mutation carries, a risk reducing salpingo-oophorectomy has been shown to reduce ovarian cancer by 85–90 % and all cause mortality as well [18–20]. Furthermore, given ongoing advances in oncology research, it is possible that by the time this future child reaches an age where he or she is at risk of malignancy, a more effective treatment or even a cure may have been found. Nevertheless, the threat of cancer may be chronically distressing for these patients, especially when considering prophylactic surgeries that carry with them a wide range of potential psychological and physical harm [3].

When asking a physician to act in accordance with the principle of non-maleficence towards a future child, the difficulty lies in defining the child’s best interest. Physicians must strive to consider objective measures whenever possible, such as extent of morbidity or mortality associated with a certain condition, when defining a child’s best interest.

### Discussion part 3

#### The physician’s duty to society: justice

Considering the broader principle of justice, encompassing the physician’s duty towards society at large, is also informative. Repeated behaviors may, over time, create medical precedents, which may eventually become accepted as standard of care. This in turn can have substantial, far-reaching public health impacts. If helping to create a life, is it therefore justifiable to mandate that that future life be as “healthy” as possible?

When invoking the justice argument, and questioning the physician’s duty towards society, it becomes pertinent to consider the notion of rationing: rationing of medical care, time, and resources. An argument can be made that the physician in our case ought to refuse to transfer an embryo that might be a BRCA-1 carrier because that future child may require extensive clinic visits, mammograms, ultrasounds, and expensive surgical interventions that could drain society’s limited resources, and that the physician should strive to create only the healthiest possible child.

Charging a physician with the task of helping to create a healthy child that will not consume disproportionate resources places a significant and unrealistic burden upon a single individual. It asks the physician to predict the needs of a future child, to assume the reaction of a future society towards supporting someone with additional needs, and to base clinical decisions on rationing unquantifiable future resources. While specific genes are increasingly becoming linked to specific disorders, knowledge of genetic inheritance patterns and epigenetic influence over genetic expression is still incomplete. Though it may be medically possible to select against a single gene or an additional chromosome in a future child’s genome, it is beyond our reach to ensure that a future child is free of all disease.

Perhaps the greatest argument against rationing is that it has the potential to threaten the physician-patient relationship. It can oppose the physician’s duty of beneficence towards the individual patient, and to the patient’s autonomy. Physicians are bound by oath to protect the well-being of their patient, and in honoring that contract, a physician’s duty to an individual patient’s care may trump that of the physician’s duty towards society.

## Conclusions

The approach to embryos with both known-carrier and indeterminate-carrier status is complex. Following its initial deliberation, the ART ethics committee requested consultation with the larger hospital ethics committee, which broadened the scope of participants to include neonatologists, medical geneticists, genetic counselors, and community members. The group agreed that, when considering the transfer or indeterminate embryos, the severity of illness and timing of onset of a disease-state should guide the clinical decision. In accordance with the ethics’ committee guidelines published by the American Society for Reproductive Medicine, adult-onset diseases are to be considered separately from childhood onset diseases [16]. As discussed, it is more ethically permissible to transfer embryos that are potential carriers for BRCA-1, a gene that confers an increased risk of a potentially non-fatal disease of adulthood, than it would be to transfer potential carriers for Tay-Sachs, a uniformly fatal disease of infancy. As outlined in the ASRM guidelines the central involvement of a genetics counselor in pre-testing planning can be extremely useful, and should be encouraged in these cases [16].

Although our ethics committee initially moved to prohibit the transfer of affected embryos, after further deliberation, this prohibition was found to be too stringent, without allowing for considerations within specific circumstances. The committee is currently creating a list of disease states in which it would recommend against the transfer of known affected embryos, which will be shared with patients.

Bringing ethical quandaries before an ethics committee relieves the burden of decision-making from the individual physician. However, the opinion of such a committee should not be a surrogate for the counseling that is an inherent part of the physician-patient relationship. While the discussion amongst professionals from different backgrounds and different areas of expertise can be enlightening, it can also over-shadow the essential role of the one-on-one relationship between a patient and her physician. The personal, therapeutic relationship between physician and patient will always foster a richer, more intricate, more subtle conversation than that had amongst a large group of people unknown to the patient herself. A benefit of this relationship is the ability to conduct an in depth informed consent process, specifically outlining all possible results. The physician can take this opportunity to create a plan with the patient regarding how to act in the case of indeterminate, affected, or non-diagnostic embryos. Prior to ART, the policies of the physician and/or clinic should be made available in writing.

Ultimately, in the case of Patient S, the physician met with the couple who agreed to proceed with transfer of the single unaffected embryo, and successfully conceived. She delivered a male infant weighing 4309 g at 40.7 weeks gestational age. The inconclusive embryos have not been re-biopsied.

As IVF and PGD become more available, greater consideration and guidance over what to do in such cases is necessary. Physicians should decide with their patients on a plan of action in advance if PGD results are inconclusive.

## References

[1] Rich TA, Liu M, Etzel CJ, Bannon SA, Mork ME, Ready K (2014). Comparison of attitudes regarding preimplantation genetic diagnosis among patients with hereditary cancer syndromes. Fam Cancer.

[2] Shenfield F, Pennings G, Devroey P, Sureau C, Tarlatzis B, Cohen J (2003). Taskforce 5: preimplantation genetic diagnosis. Hum Reprod.

[3] Derks-Smeets IAP, Gietel-Habets JJG, Tibben A, Tjan-Heijnen VCG, Meijer-Hoogeveen M, Geraedts JPM (2014). Decision-making on preimplantation genetic diagnosis and prenatal diagnosis: challenge for couples with hereditary breast and ovarian cancer. Hum Reprod.

[4] Chang LJ, Huang CC, Tsai YY, Hung CC, Fang MY, Lin YC (2013). Blastocyst biopsy and vitrification are effective for preimplantation genetic diagnosis of monogenic diseases. Hum Reprod.

[5] Harper JC, Wilton L, Traeger-Synodinos J, Goossens V, Moutou C, SenGupta SB (2012). The ESHRE PGD Consortium: 10 years of data collection. Hum Reprod Update.

[6] Wilton L, Thornhill A, Traeger-Synodinos J, Sermon KD, Harper JC (2009). T*he causes of misdiagnosis and adverse outcomes in PGD*. Hum Reprod.

[7] Colls P, Escudero T, Cekleniak N, Sadowy S, Cohen J, Munné S (2007). Increased efficiency of preimplantation genetic diagnosis for infertility using "no result rescue.". Fertil Steril..

[8] Beauchamp TL, Childress JF. Principles of Biomedical Ethics, 6th edition. New York: Oxford University Press. 2008.

[9] Chen S, Parmigiani G (2007). Meta-analysis of BRCA1 and BRCA2 penetrance. J Clin Oncol.

[10] Euhus DM, Robinson L (2013). Genetic predisposition syndromes and their management. Surgical Clinics of North America.

[11] Taylor TH, Patrick JL, Gitlin SA, Michael Wilson J, Crain JL, Griffin DK (2014). Outcomes of blastocysts biopsied and vitrified once versus those cryopreserved twice for euploid blastocyst transfer. Reprod Biomed Online.

[12] Koch J, Costello MF, Chapman MG, Kilani S (2011). Twice-frozen embryos are no detriment to pregnancy success: a retrospective comparative study. Fertil Steril.

[13] de Wert G, Dondorp W, Shenfield F, Devroey P, Tarlatzis B, Barri P (2014). ESHRE Task Force on Ethics and Law22: Preimplantation Genetic Diagnosis. Hum Reprod.

[14] Pennings G, de Wert G, Shenfield F, Cohen J, Tarlatzis B, Devroey P (2007). ESHRE Task Force on Ethics and Law 13: the welfare of the child in medically assisted reproduction. Hum Reprod.

[15] McArthur SJ, Leigh D, Marshall JT, de Boer KA, Jansen RP (2005). Pregnancies and live births after trophectoderm biopsy and preimplantation genetic testing of human blastocysts. Fertil Steril.

[16] Ethics Committee of American Society for Reproductive Medicine (2013). Use of preimplantation genetic diagnosis for serious adult onset conditions: a committee opinion. Fertil Steril..

[17] Baruch S, Kaufman D, Hudson KL (2008). Genetic testing of embryos: practices and perspectives of US in vitro fertilization clinics. Fertil Steril.

[18] Domchek SM, Friebel TM, Singer CF, Evans DG, Lynch HT, Isaacs C (2010). Association of risk-reducing surgery in BRCA1 or BRCA2 mutation carriers with cancer risk and mortality. JAMA.

[19] Domchek SM, Friebel TM, Neuhausen SL, Wagner T, Evans G, Isaacs C (2006). Mortality after bilateral salpingo-oophorectomy in BRCA1 and BRCA2 mutation carriers: a prospective cohort study. Lancet Oncol.

[20] Garcia C, Wendt J, Lyon L, Jones J, Littell RD, Armstrong MA (2014). Risk management options elected by women after testing positive for a BRCA mutation. Gynecol Oncol.

